# Clusters of Internally Primed Transcripts Reveal Novel Long Noncoding RNAs

**DOI:** 10.1371/journal.pgen.0020037

**Published:** 2006-04-28

**Authors:** Masaaki Furuno, Ken C Pang, Noriko Ninomiya, Shiro Fukuda, Martin C Frith, Carol Bult, Chikatoshi Kai, Jun Kawai, Piero Carninci, Yoshihide Hayashizaki, John S Mattick, Harukazu Suzuki

**Affiliations:** 1 Mouse Genome Informatics Consortium, The Jackson Laboratory, Bar Harbor, Maine, United States of America; 2 Australian Research Council Special Research Centre for Functional and Applied Genomics, Institute for Molecular Bioscience, University of Queensland, Brisbane, Australia; 3 T Cell laboratory, Ludwig Institute for Cancer Research, Austin Health, Heidelberg, Victoria, Australia; 4 Genome Exploration Research Group (Genome Network Project Core Group), RIKEN Genomic Sciences Center, RIKEN Yokohama Institute, Yokohama, Japan; 5 Genome Science Laboratory, Discovery Research Institute, RIKEN Wako Institute, Wako, Japan; The Jackson Laboratory, US; MRC-Harwell, UK; NHGRI-NIH, US; Lawrence Livermore National Laboratory, US; The Jackson Laboratory, US

## Abstract

Non-protein-coding RNAs (ncRNAs) are increasingly being recognized as having important regulatory roles. Although much recent attention has focused on tiny 22- to 25-nucleotide microRNAs, several functional ncRNAs are orders of magnitude larger in size. Examples of such macro ncRNAs include *Xist* and *Air,* which in mouse are 18 and 108 kilobases (Kb), respectively. We surveyed the 102,801 FANTOM3 mouse cDNA clones and found that *Air* and *Xist* were present not as single, full-length transcripts but as a cluster of multiple, shorter cDNAs, which were unspliced, had little coding potential, and were most likely primed from internal adenine-rich regions within longer parental transcripts. We therefore conducted a genome-wide search for regional clusters of such cDNAs to find novel macro ncRNA candidates. Sixty-six regions were identified, each of which mapped outside known protein-coding loci and which had a mean length of 92 Kb. We detected several known long ncRNAs within these regions, supporting the basic rationale of our approach. In silico analysis showed that many regions had evidence of imprinting and/or antisense transcription. These regions were significantly associated with microRNAs and transcripts from the central nervous system. We selected eight novel regions for experimental validation by northern blot and RT-PCR and found that the majority represent previously unrecognized noncoding transcripts that are at least 10 Kb in size and predominantly localized in the nucleus. Taken together, the data not only identify multiple new ncRNAs but also suggest the existence of many more macro ncRNAs like *Xist* and *Air*.

## Synopsis

The human genome has been sequenced, and, intriguingly, less than 2% specifies the information for the basic protein building blocks of our bodies. So, what does the other 98% do? It now appears that the mammalian genome also specifies the instructions for many previously undiscovered “non protein-coding RNA” (ncRNA) genes. However, what these ncRNAs do is largely unknown. In recent years, strategies have been designed that have successfully identified hundreds of short ncRNAs—termed microRNAs—many of which have since been shown to act as genetic regulators. Also known to be functionally important are a handful of ncRNAs orders of magnitude larger in size than microRNAs. The availability of complete genome and comprehensive transcript sequences allows for the systematic discovery of more large ncRNAs. The authors developed a computational strategy to screen the mouse genome and identify large ncRNAs. They detected existing large ncRNAs, thus validating their approach, but, more importantly, discovered more than 60 other candidates, some of which were subsequently confirmed experimentally. This work opens the door to a virtually unexplored world of large ncRNAs and beckons future experimental work to define the cellular functions of these molecules.

## Introduction

The existence of non-protein-coding RNAs (ncRNAs) has been known for many decades, and the importance of essential infrastructural ncRNAs such as ribosomal RNAs and transfer RNAs in facilitating protein synthesis has long been recognized. Recently, other ncRNAs have generated intense interest based upon their ability to regulate gene expression. Foremost among these are microRNAs (miRNAs), which are about 22 nucleotides in length and function by targeting mRNAs for cleavage or translational repression. Hundreds of miRNAs have been identified in animals, plants, and viruses, and they mediate critical regulatory functions in a range of developmental and physiological pathways [1–3]. Another prominent class of ncRNAs is the short interfering RNAs (siRNAs), which were discovered as a tool for knocking down gene expression in the lab but have subsequently been found to act as natural endogenous regulators of gene expression [1].

Given the considerable attention that these tiny ncRNAs have attracted, it would be understandable to think that regulatory ncRNAs are short. However, a small number of functional ncRNAs have also been identified that are orders of magnitude larger in size than miRNAs and siRNAs. Well-known examples of such macro ncRNAs include *Xist* and *Air,* which in mouse are approximately 18 and 108 Kb, respectively [4,5]. *Xist* plays an essential role in mammals by associating with chromatin and causing widespread gene silencing on the inactive X chromosome [6], while *Air* is required for paternal silencing of the *Igf2r/Slc22a2/Slc22a3* gene cluster [5]. Apart from their extreme length, *Xist* and *Air* share two other important features: genomic imprinting and antisense transcription. Genomic imprinting is a process by which certain genes are expressed differently according to whether they have been inherited from the maternal or paternal allele. Imprinting is critical for normal development, and loss of imprinting has been implicated in a variety of human diseases [7]. ncRNAs have been discovered at many different imprinted loci and appear to be important in the imprinting process itself [5,8]. The other feature that *Xist* and *Air* have in common is that both are members of naturally occurring *cis*-antisense transcript pairs. Previous studies have indicated the existence of thousands of mammalian *cis*-antisense transcripts [9–12]. These transcripts may regulate gene expression in a variety of ways including RNA interference, translational regulation, RNA editing, alternative splicing, and alternative polyadenylation [13,14], although the exact mechanisms by which antisense RNAs function are unknown.

In addition to well-documented ncRNAs, recent evidence from both high-density tiling arrays [15,16] and large-scale analyses of full-length enriched cDNA libraries [17] suggests that there may be thousands more ncRNAs within the mammalian transcriptome. Many of these candidates have emerged from the RIKEN Mouse Gene Encyclopedia project [17,18], and full-length sequencing and analysis by the FANTOM consortium of 102,801 cDNAs recently revealed that around one-third (34,030) lack an apparent protein-coding region as judged by manual annotation [19]. Although some of these RNAs have been shown to have biological function [20,21], the vast majority of these putative noncoding cDNAs remain of uncertain significance, especially given that many are likely to represent internally primed transcription artifacts (which arise during first-strand cDNA synthesis when oligo[dT] primers bind not to genuine polyA tails but rather to internal adenine-rich regions within longer transcripts) and are not true, full-length transcripts [22,23].

In surveying the FANTOM3 mouse cDNAs, we observed that macro ncRNAs such as *Air* and *Xist* were present not as single, full-length transcripts but rather as fragmented clusters of cDNAs, most of which were not only internally primed but also unspliced and of minimal protein-coding potential. We hypothesized that we might discover novel macro ncRNAs by conducting a genome-wide search for similar clusters of cDNAs. We subsequently identified 66 candidate ncRNA regions. A few of these overlap with known long ncRNAs, and many contain imprinted cDNA candidates, *cis*-antisense transcripts, or miRNAs. Eight regions were characterized experimentally, and the majority were found to represent previously unknown long ncRNAs that are localized to the nucleus. Taken together, the data suggest the existence of many more macro ncRNAs that, like *Xist* and *Air,* may fulfill important regulatory roles in mammalian biology.

## Results

### *Xist* and *Air* Are Represented by Clusters of Truncated Noncoding cDNAs

**Figure 1:**
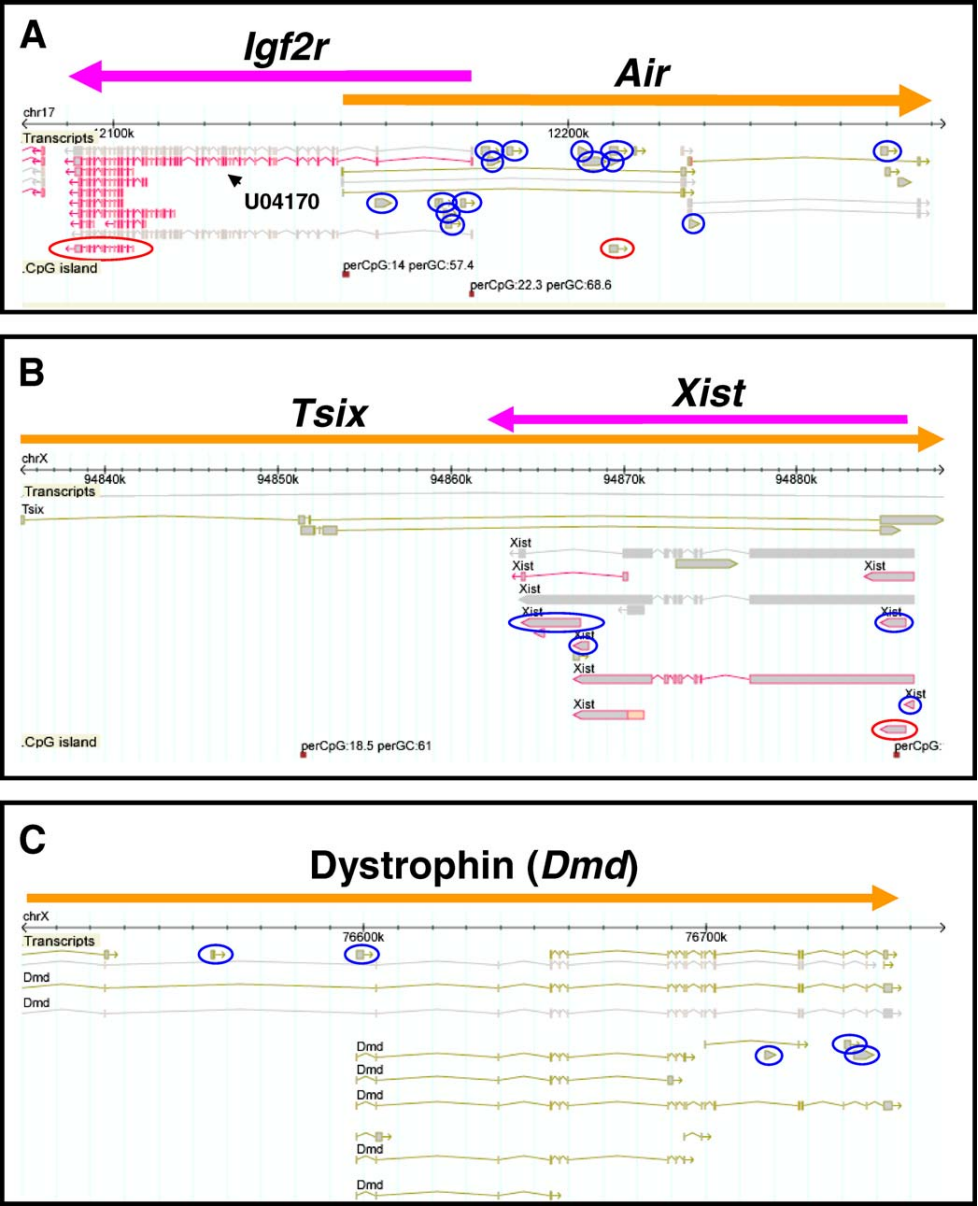
Snapshots of the GEV Showing Transcription (A) The *Air*/*Igf2r* locus (Chromosome 17: 12,091,531–12,258,195). (B) The *Xist*/*Tsix* locus (X chromosome: 94,835,096–94,888,536). (C) The dystrophin *(Dmd)* locus (X chromosome: 76,500,000–76,754,601). For the transcripts, cDNA sequences from the RIKEN and public databases are shown, and are colored in brown and purple depending upon their chromosomal strand of origin. Predicted genes from Ensembl, NCBI, and RefSeq databases are shown in gray. CpG islands as defined by the UCSC Genome Browser are shown. Blue circles indicate unspliced, noncoding RIKEN cDNAs with adjunct adenine-rich regions. Red circles indicate RIKEN imprinted cDNA candidates [38].

As part of the FANTOM3 project, we looked for the existence of known ncRNAs among the 102,801 cDNAs. We found that 16 of 43 (39%) non-small-nucleolar, non-micro reference mouse ncRNAs that are present in RNAdb, a database of mammalian ncRNAs [24], were detectable among the RIKEN cDNA collection, as judged by similarity using BLASTN (Table 1). The two longest ncRNAs detected were *Xist* and *Air*. Very long transcripts such as these create substantial difficulties for cDNA cloning protocols for a variety of well-established technical reasons [23,25]. We were therefore not surprised that examination of both loci via the FANTOM3 Genomic Element Viewer (GEV) (http://fantom32p.gsc.riken.jp/gev-f3/gbrowse/mm5) revealed that *Xist* and *Air* were represented by a cluster of truncated RIKEN and non-RIKEN cDNAs interspersed along the length of their parent transcripts. Inspection of the individual cDNAs demonstrated that the majority were unspliced, held minimal protein-coding potential, and had adjunct genomic adenine-rich regions immediately downstream of their 3′ ends, suggesting that they had been internally primed. Figure 1A illustrates transcription within the *Air/Igf2r* locus. *Air* is represented by 20 individual cDNAs dispersed along its reported length, of which 14 are unspliced, noncoding RIKEN cDNAs that contain an adjunct adenine-rich region. Figure 1B shows *Xist* and its antisense partner *Tsix*. Here, nine cDNAs are seen along the length of the spliced *Xist* transcript, of which four are unspliced, noncoding RIKEN cDNAs that contain an adjunct adenine-rich region.

**Table 1:**
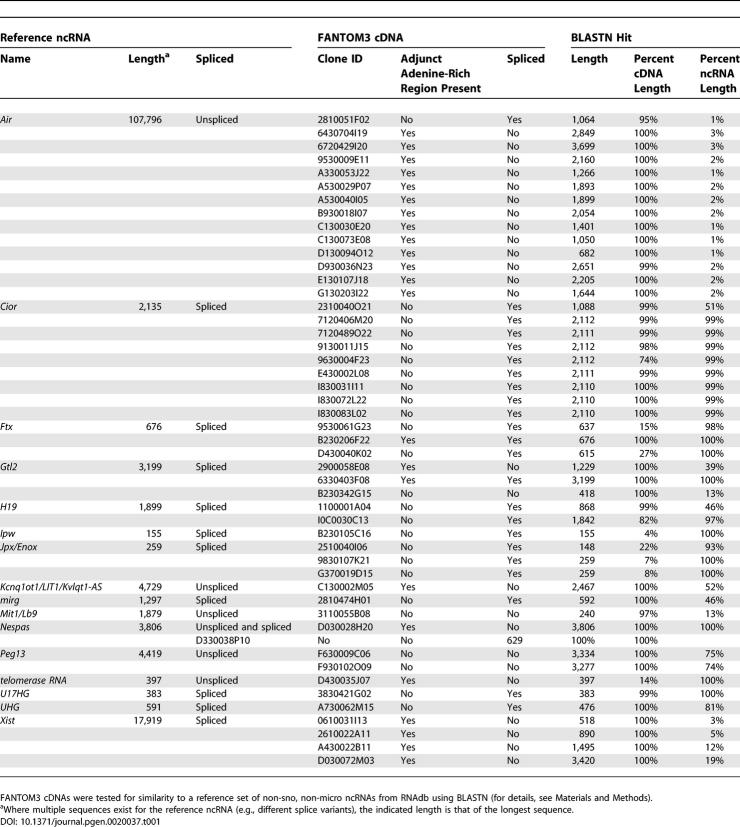
Detection of Known Mouse ncRNAs within the FANTOM3 cDNA Collection

### Genome-Wide Search Reveals Multiple Clusters of Unspliced, Internally Primed Noncoding Transcripts Lying Outside Protein-Coding Loci

**Figure 2:**
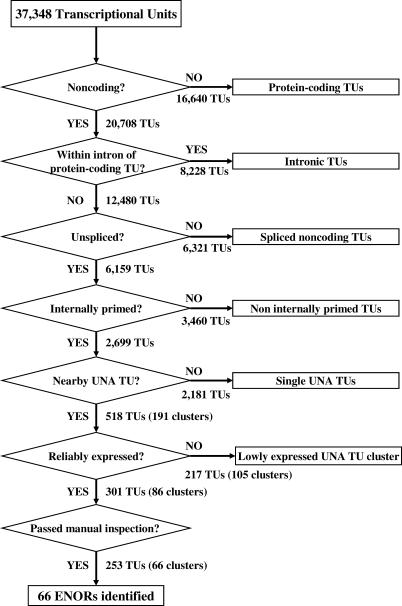
Discovery Pipeline for ENORs FANTOM and public transcripts were clustered into 37,348 TUs by grouping any two or more transcripts that shared genomic coordinates. Then, the following procedures were applied. (1) Protein-coding TUs were excluded by removing any whose transcripts had an open reading frame of either 150 amino acids or more (RIKEN/MGC cDNAs) or one amino acid or more (non-RIKEN/MGC cDNAs). (2) TUs wholly encompassed within introns of protein-coding TUs were excluded to avoid possible pre-mRNA intronic transcripts. (3) Intron-containing TUs were excluded to select for unspliced transcripts. (4) TUs lacking adjunct adenine-rich regions or containing polyA signals were excluded to select for internally primed transcripts. (5) Remaining UNA TUs that mapped within 100 Kb of one another on the mouse genome (mm5) were clustered together, provided they did not overlap the genomic coordinates of a protein-coding TU/NCBI RefSeq/Ensembl gene model with a CDS of 150 amino acids or more or a noncoding TU with a polyA signal within 100 bp of the 3′ end and without an adjunct adenine-rich region. (6) Reliably expressed UNA TU clusters were selected by identifying those with at least ten supporting ESTs. (7) Selected UNA TU clusters were then manually screened and separated based upon evidence of possible internal transcription state sites (based upon CpG islands, CAGE tags, and EST clusters), resulting in the identification of 66 ENORs.

Based upon these observations (Table 1; Figure 1), we reasoned that it might be possible to discover novel macro ncRNAs via a genome-wide search for clusters of transcripts that were unspliced, noncoding, and contained adjunct adenine-rich regions (UNA transcripts) (Figure 2). To begin, we classified transcriptional units (TUs) into protein-coding and noncoding using the manual annotations of FANTOM3 collaborators [19], where a TU is defined as a group of transcripts that share at least one exonic nucleotide overlap and that map to the same chromosomal strand [19]. Of 37,348 TUs, 20,708 were classified as noncoding TUs. We knew, however, from previous work that noncoding TUs often overlap with protein-coding genes, since they can be internally primed off long pre-mRNAs [22]. Figure 1C shows an example of this, where a cluster of five UNA cDNAs overlap with intronic regions of the large dystrophin *(Dmd)* transcript. Of 20,708 noncoding TUs, we excluded 8,228 located within intronic regions of protein-coding TUs. We then selected UNA TUs based on the following criteria: (1) an adjunct adenine-rich region was present at the TU end, (2) no major polyA signal (AATAAA/ATTAAA) was present within 100 nucleotides of the TU end, and (3) the TU was unspliced. Of 12,480 noncoding TUs, 2,699 satisfied the criteria. We then clustered these 2,699 UNA TUs by merging any two or more located within 100 Kb of one another, provided that (1) there were no intervening protein-coding transcripts or gene predictions (based on either FANTOM3 annotations, NCBI RefSeq sequences, or Ensembl gene models) and (2) there were no intervening transcripts with major polyA signals and without adjunct adenine-rich regions (which would indicate likely transcript termination sites). Using this approach, we identified 191 genomic regions, containing 528 clustered UNA TUs. To increase the likelihood that these regions represented genuine long transcripts, we excluded any that were less than 10 Kb long or contained less than ten expressed sequence tags (ESTs). This left 86 regions, which were then manually inspected using the GEV to look for possible internal transcriptional start sites (e.g., CpG islands, CAGE tags, or multiple ESTs arising from the same position) or transcripts encoding small proteins not already filtered out in the discovery pipeline. Following this, 66 regions remained (Table 2). We named these long expressed noncoding regions (ENORs).

**Table 2:**
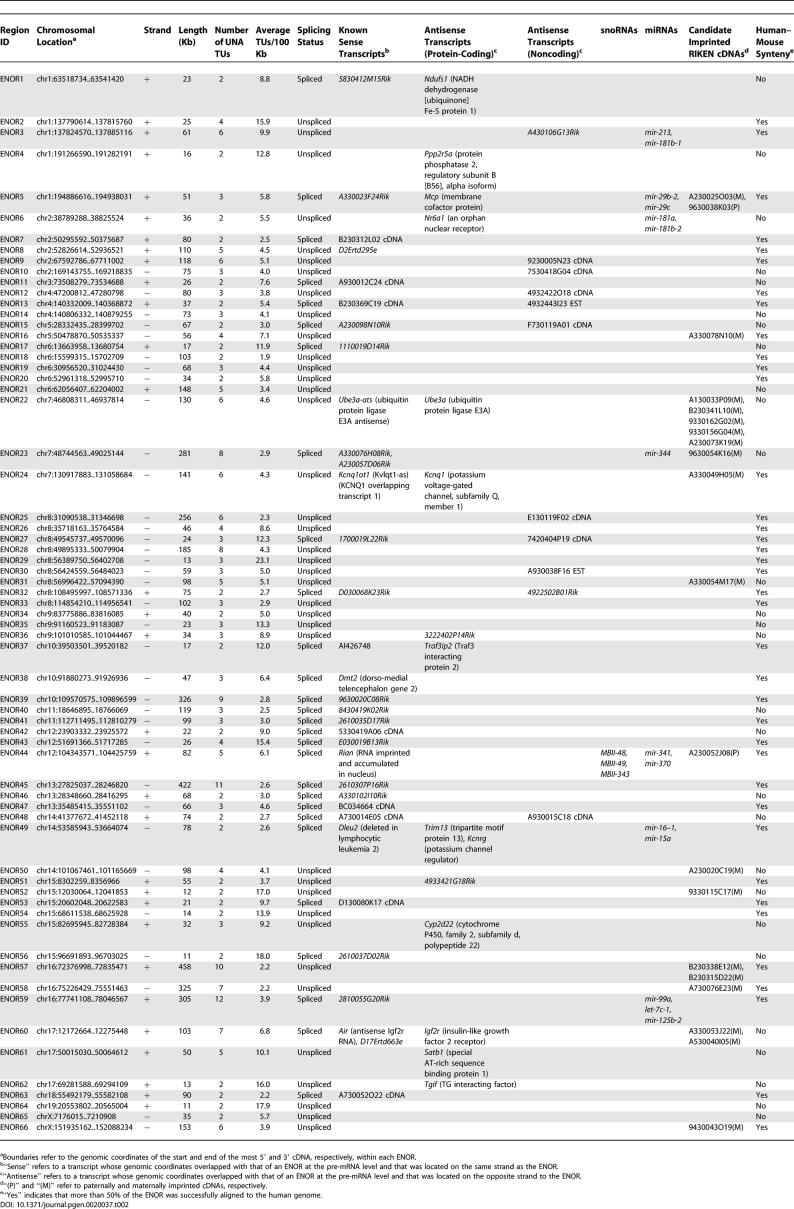
Bioinformatic Characterization of 66 ENORs

### ENORs Successfully Identify Several Known Long ncRNAs

To assess the validity of our approach, we examined whether known mouse macro ncRNAs were detected among the 66 ENORs. Notably, the cluster we had manually identified as corresponding to *Xist* was not detected. This was because one of the original *Xist* transcripts (GenBank accession number X59289) remains annotated as a hypothetical protein of 299 amino acids based upon an earlier presumption that it was translated [26]; consequently, this cluster of cDNAs was automatically classified as being protein-coding and thus rejected. We did, however, succeed in identifying *Air* (ENOR60) and several other long ncRNAs. These included the following: *Kcnq1ot1* (ENOR24), an imprinted antisense transcript of ~54 Kb [27]; *Rian* (ENOR44), a spliced 5.4-Kb imprinted transcript that spans more than 10 Kb of mouse genome and acts as a host gene for multiple small nucleolar RNAs (snoRNAs) [28,29]; and *Ube3a-ats* (ENOR22), an imprinted, ~1,000-Kb antisense transcript that is brain-specific and hosts numerous snoRNAs [30]. Additionally, we detected *Dleu2* (ENOR49), an alternatively spliced antisense ncRNA of ~1.4 Kb that spans more than 80 Kb and is a host gene for miRNAs [31,32]. Apart from *Xist,* the only other ncRNA in the RNAdb reference set longer than 5 Kb that was not detected was *Emx2os,* a 5.04-Kb antisense transcript that spans ~35 Kb [33]. Inspection of this locus showed that it contained only one UNA cDNA. Taken together, these observations indicated that our approach was able to successfully detect existing long ncRNAs, although it missed some either because of annotation errors or because the number of UNA transcripts fell below our discovery pipeline threshold. Our approach also appeared to detect shorter ncRNAs such as *Dleu2* that were spliced and spanned a long genomic region.

### In Silico Characterization of ENOR Regions

Next, we sought to characterize the 66 ENORs in greater detail (Table 2). The maximum number of UNA TUs per region was 12 (ENOR59), and the average was 3.8 per 100 Kb. The region length ranged from 11 to 458 Kb, with a mean of 92 Kb. The number, length, and distribution of the ENORs across each chromosome are shown in Table S1 and Figure S1. Chromosome 8 had the highest number of ENORs (nine), with a total length of 860 Kb. Chromosome 16 had the greatest length (1,089 Kb), as represented by three ENORs. The total length of the 66 ENORs was 6,044 Kb, corresponding to 0.23% of the mouse genome.

We classified the 66 ENORs based upon the frequency of spliced and unspliced ESTs (Table 3). Twenty-eight regions contained numerous spliced ESTs, while the remaining 38 regions included no or very few spliced ESTs. The longest unspliced region was ENOR57, which included ten UNA TUs spanning almost 460 Kb. Interestingly, we found that *Air,* which has previously been reported as unspliced [5], overlapped with several spliced cDNAs and ESTs, suggesting that *Air* may also exist as spliced isoforms. Consistent with this idea, there is another ncRNA, *Nespas,* for which multiple spliced and unspliced forms have been reported, and the human–mouse conservation of these different isoforms suggests that they may be functionally relevant [34].

**Table 3:**
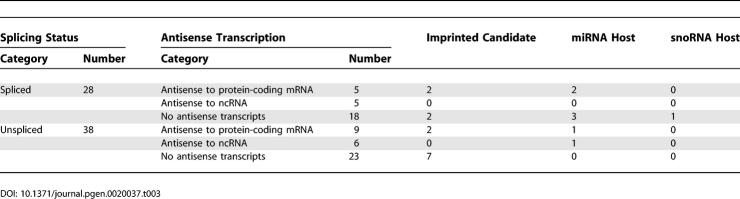
Summary Characteristics of 66 ENORs

Sequence conservation between different species indirectly suggests function. To assess the conservation of the ENORs, we searched for syntenic human loci using mouse–human whole genome alignments available from the University of California Santa Cruz (UCSC) Genome Browser [35,36]. Many ENORs (38 of 66) could be successfully aligned between mouse and human over at least 50% of their length (Table 2). However, a significant minority were not well-conserved, and these included known functional ncRNAs such as *Air* and *Ube3a-ats,* which highlights that a lack of conservation does not necessarily imply a lack of function [37]. Because some long poorly conserved ncRNAs such as *Xist* retain patches of well-conserved sequence [6], we also examined ENOR conservation in short 50-nucleotide windows (Figure S2). This approach indicated not only that ENORs have patches of high conservation but also that they are more conserved than the genome average, so that while only ~45% of the mouse genome windows are alignable to the human genome, ~60% of ENOR windows are alignable.

### ENORs Show Evidence of Imprinting and Antisense Transcription

Because previous studies revealed associations between macro ncRNAs and both imprinting and antisense transcription, we looked to see if our ENOR loci were associated with either of these phenomena.

To examine imprinting, we obtained 2,114 candidate imprinted mouse transcripts previously identified by Nikaido et al. [38]. By mapping these transcripts to the mouse genome (May 2004 assembly; mm5), we found that 13 ENORs (containing 20 candidate imprinted cDNAs) showed evidence of imprinting (Tables 2 and 3). This number was significantly higher than expected by chance (Chi-square, *p* < 0.001). Of the 13 ENORs identified, four contained well-characterized imprinted ncRNAs *(Rian, Air, Ube3a-ats,* and *Kcnq1ot1)* and nine represent potentially imprinted ncRNAs.

To characterize *cis*-antisense transcription, we searched for transcripts that appeared in the complementary strand of each ENOR (Tables 2 and 3). Of 28 spliced ENORs, two corresponded to known antisense ncRNAs *(Air* and *Dleu2),* and a further eight represented potentially novel antisense transcripts to either protein-coding genes *(Mcp, Ndufs1,* and *Traf3ip2)* or to noncoding transcripts. In the case of *Dleu2,* which has been suggested to play a role in the splice-site regulation of its cognate antisense partner *Trim13* [32], we also identified a potentially new antisense partner, *Kcnrg*. Of 38 unspliced ENORs, two corresponded to known ncRNAs antisense to *Ube3a* and *Kcnq1,* and a further 13 represented potentially novel antisense transcripts to either protein-coding genes *(Cyp2d22, Nr6a1, Ppp2r5a, Satb1, Tgif*, *3222402P14Rik,* and *4933421G18Rik)* or to noncoding transcripts. Many of the protein-coding genes are involved in development and disease, and, as with *Igf2r* and *Air,* the discovery of long noncoding antisense transcripts may be very important in understanding the regulation of these genes.

### ENORs Are Associated with miRNAs and Show Tissue-Specific Expression

As indicated above, a number of ENORs corresponded to known ncRNAs that act as host genes for either snoRNAs or miRNAs. We were therefore interested to see whether any other ENORs contained miRNAs or snoRNAs. We downloaded 224 known miRNAs and 175 snoRNAs from the miRBase Registry and RNAdb, respectively [24,39]. We then mapped these sequences to the mouse genome, and examined them for overlap with the 66 ENORs. We found that seven ENORs overlapped with 14 known miRNAs (14/224; 6%; Table 2), an association unlikely to have occurred by chance (*p* < 0.0001). Some of these ENORs also contained imprinted cDNA candidates, in keeping with a previously noted association between miRNAs and imprinting [40]. No new snoRNA hosts were found.

**Figure 3:**
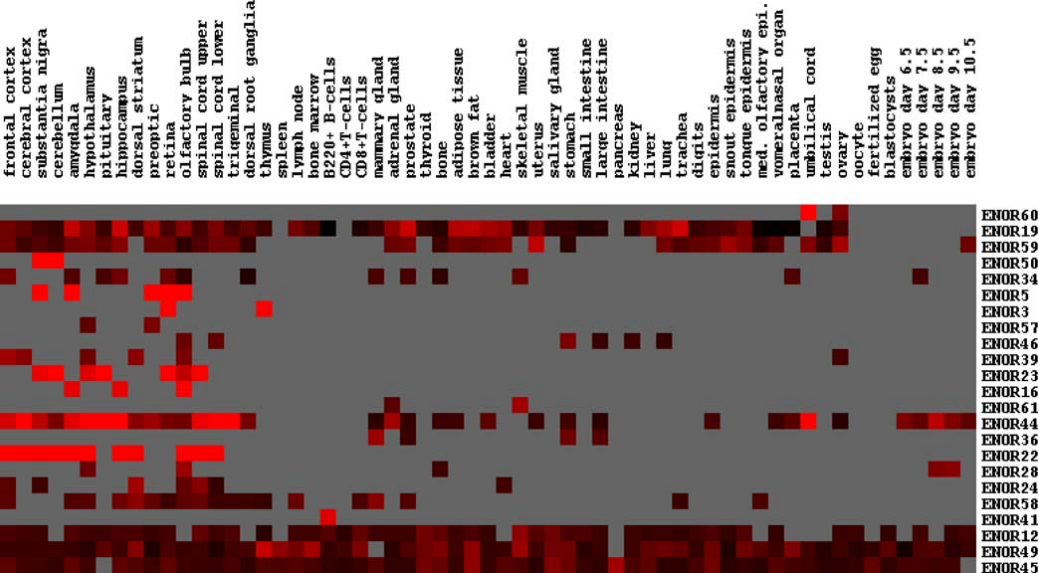
ENOR Tissue Expression Tissue expression information for individual ENORs was obtained using publicly available GNF Gene Expression Atlas data. GNF probes that overlapped ENORs were identified, and the corresponding relative expression ratios for 61 tissues were hierarchically clustered. Red squares indicate high expression, black squares indicate low expression, and grey squares indicate where expression was not reliably detected (based upon Affymetrix MAS5 absent/present calls). med. olfactory epi., medial olfactory epithelium.

Next, we examined the expression of ENOR transcripts. Using the publicly available mouse gene expression atlas data from the Genomics Institute of the Novartis Research Foundation (GNF) [41], we found that 23 ENORs were expressed in at least one of the 61 tissues examined. Thirty-three of the remaining ENORs did not have any corresponding GNF probes, while a further ten had probes whose expression was not reliably detected. Of the 23 ENORs, some were expressed almost ubiquitously, while others showed a restricted, tissue-specific expression profile (Figure 3). Notably, many ENORs were enriched in the central nervous system (CNS), and these included known brain-specific ncRNAs *Ube3a-ats* (ENOR22) and *Rian* (ENOR44) [28,30]. Because only a minority of ENORs had supporting GNF information, we also used RIKEN EST data to assess whether ENOR transcripts showed preferential expression in particular tissues. We searched for RIKEN ESTs that mapped within each ENOR and tallied the number of clones associated with the ESTs that were derived from a particular tissue (as per Edinburgh Mouse Atlas Project descriptions), and then we compared these counts with those of the entire FANTOM3 set. We found that ENOR transcripts as a whole are significantly overrepresented in a number of tissues including the CNS (Table S2). A caveat to this result is that RIKEN ESTs were derived after intensive subtraction, and their relative abundance might therefore not reflect natural tissue expression, although the EST data were in general agreement with the GNF results for a number of ENORs.

As noted earlier, spliced ENORs such as that corresponding to *Dleu2* may not necessarily represent macro ncRNAs because clusters of UNA transcripts may be derived from the introns of longer pre-mRNAs whose final product may be less than 10 Kb. To proceed, we therefore focused our attention on the unspliced class of ENORs, which we reasoned were most likely to represent novel macro ncRNAs.

### Long PCR and Quantitative RT-PCR Provide Indirect Evidence of Macro ncRNAs

**Figure 4:**
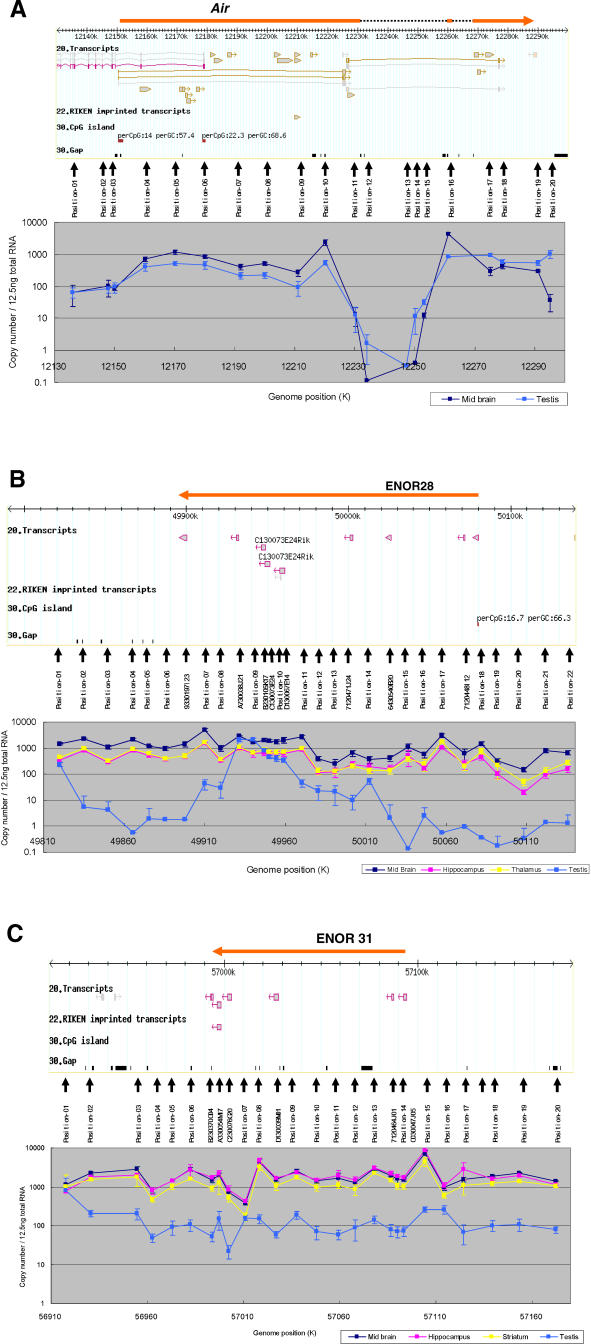
qRT-PCR Analysis Analysis of (A) *Air,* (B) ENOR28, and (C) ENOR31 loci. Above in each panel, screen shots of the GEV featuring the loci around *Air,* ENOR28, and ENOR31 are shown. The orange bars indicate the regions for *Air,* ENOR28, and ENOR31. cDNA sequences from the RIKEN and public databases are shown. Sequences mapped on the plus strand and minus strand are brown and purple, respectively. Predicted genes from Ensembl, NCBI, and RefSeq databases are shown in gray. For RIKEN imprinted transcripts, imprinted cDNA candidates identified previously [38] are shown. CpG islands as defined by the UCSC Genome Browser are shown. Positions of primer pairs are marked by small vertical arrows. Below in each panel, qRT-PCR results for midbrain, hippocampus, thalamus, striatum, and testis using the corresponding primer pairs are shown.

As proof of principle, we selected two regions for initial experimental characterization: ENOR28 and ENOR31 (Figure 4). ENOR28 (Figure 4B) was located on Chromosome 8 (49,895,333–50,079,904; mm5), appeared unspliced, spanned 185 Kb, and contained eight UNA TUs. ENOR31 (Figure 4C) was also on Chromosome 8 (56,996,422–57,094,390), appeared unspliced, spanned 98 Kb, and contained five UNA TUs, one of which was a possible imprinted transcript [38]. The majority of cDNAs in both regions were from common tissues (CNS), and this—together with their lack of splicing and greater than average length—made them good initial candidates.

**Figure 5:**
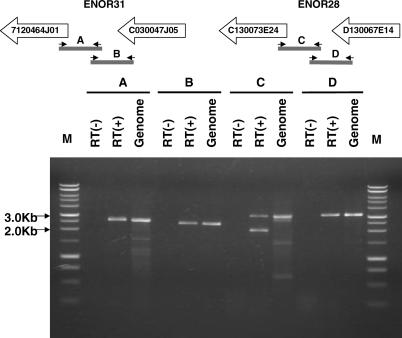
Presence of Transcription between Adjacent cDNAs PCR was carried out with and without reverse transcription (RT[+] and RT[−], respectively) using midbrain total RNA and the corresponding primer pairs (see Table S3). PCR using genomic DNA was also carried out as a control. A DNA ladder (Promega; http://www.promega.com) was used as a size marker. The amplified fragments were confirmed as the expected ones by analyzing digestion pattern using several restriction enzymes. The lower band, observed in the RT(+) lane of the amplified fragment C, seems to be nonspecific, because it was amplified using only the right primer and because it showed a digestion pattern with restriction enzymes quite different from that of the upper band and the band of the genomic DNA (unpublished data).

Initially, we looked for the presence of transcription between neighboring cDNAs by long PCR (all of the PCR primers are shown in Table S3). Figure 5 shows that we successfully amplified transcripts between cDNAs 7120464J01 and C030047J05 in ENOR31, and between cDNAs C130073E24 and D130067E14 in ENOR28. These results suggested that directly adjacent cDNAs arise from a common transcript.

If each region represents one continuous transcript under the control of a single promoter in a given tissue, we reasoned, then across multiple tissues the levels of expression for each ENOR cDNA should remain consistent with those of the other cDNAs in the region. Total RNA was therefore isolated from eight different tissues, and each ENOR cDNA expression profile was examined by quantitative real-time RT-PCR (qRT-PCR). We found that the expression of all cDNAs (apart from A730038J21 in ENOR28) was highly intercorrelated (*R* > 0.9; Table S4), thus providing indirect evidence that not only directly adjacent cDNAs but also those more remote from one another were from the same transcript.

### Northern Blots Directly Confirm Existence of Multiple Novel Macro ncRNAs

**Figure 6:**
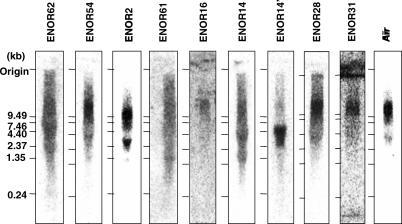
Northern Blot Analysis of ENOR Transcripts Mouse whole brain total RNA (10 μg/lane) was used for the analysis except for ENOR2 and ENOR61, where mouse thymus total RNA was used. DNA fragments without any predicted repeated sequences were PCR-amplified from cDNAs in ENORs (Table S3), labeled with ^32^P-dCTP (Amersham Biosciences), and then used as probes. RNA size was estimated with an RNA ladder (Invitrogen). ENORs are listed in increasing order based on the estimated length of each region.

Northern blot analysis is a direct means to demonstrate the existence of very large RNAs. We therefore selected eight ENORs (ENOR2, ENOR14, ENOR16, ENOR28, ENOR31, ENOR54, ENOR61, and ENOR62), which together were representative of a broad range of lengths, chromosomes, and EST abundance, and tested them by northern blot using specific probes (Table S3). As a positive control, we also examined ENOR60, which corresponds to *Air*. Figure 6 shows that *Air* was readily detected as a band greater than 10 Kb in size. Similarly, probes against ENOR2, ENOR16, ENOR28, ENOR31, and ENOR54 all detected clearly visible bands greater than 10 Kb. Other ENORs gave less clear results. ENOR61 had a broad signal that appeared as a smear originating from the upper reaches of the gel, and it was unclear whether this was due to degradation of a large transcript or was nonspecific. ENOR62 produced a similar result. Probes for ENOR14, on the other hand, detected one major product larger than 7.5 Kb and possibly another larger than 10 Kb. Thus, in six of nine cases, we were able to successfully demonstrate macro RNAs larger than 10 Kb and in the remaining three cases the results were equivocal.

### Detailed qRT-PCR Analysis Reveals That ENORs Might Contain Multiple Long Transcripts

Northern blots do not accurately resolve the size of transcripts larger than 10 Kb. For this reason, the 108-Kb *Air* transcript in Figure 6 appears only just above 10 Kb (which is similar to the original northern blot result obtained by Lyle et al. [42]), and the actual size of the other macro ncRNAs cannot be successfully determined from the blots. To gain a better understanding of the true extent of transcription across our regions, we therefore performed further qRT-PCR analysis across our original candidate ENORs, ENOR28 and ENOR31. Specific primer pairs were designed before, after, and along the length of the region, incorporating individual cDNAs as well as the areas in between (Figure 4B and 4C; Table S3). As a control, ENOR60, containing *Air,* was analyzed in a similar manner (Figure 4A; Table S3).

To begin, we extracted total RNA from different CNS tissues (midbrain, hippocampus, corpus striatum, and thalamus) and from testis, and assessed the level of expression of at least 20 separate subregions spanning the length of both ENORs as well as *Air*. Figure 4A demonstrates that, in the *Air* locus, expression arises from downstream of a CpG island, as previously reported [43], and then remains relatively constant for the next 70–80 Kb. Beyond this, expression falls below 100 transcripts per 12.5 ng of total RNA for the next 30–40 Kb (primer pairs 11–15) then rises and plateaus again for a further 30 Kb. Examination of the alignment between the genomic DNA sequence of *Air* (GenBank accession number AJ249895) and the genome assembly revealed that there are two inserted sequences in the genome assembly (dotted lines in Figure 4A). These sequences are disconnected by gaps in the genome assembly, indicating that the transient fall in expression is an artifact. Overall, then, this result was in keeping with the presence of a continuous macro ncRNA ~100–110 Kb in size, and provided evidence that the qRT-PCR-based strategy employed here was able to successfully detect such long transcripts and to provide a reasonable estimate of their size.

Figure 4B illustrates expression across the ENOR28 locus. In CNS tissues, the overall expression pattern was similar to that of *Air,* with sustained expression over tens of kilobases at transcript copy numbers in the hundreds to thousands (per 12.5 ng of total RNA). Looking more closely and starting from upstream of the 5′ end, expression levels are at their lowest around primer pair 20, dipping below 100 copies; next, transcript levels for primer pairs 12–18 are intermediate; finally, from primer pairs 1–11 (a distance larger than 100 Kb), expression is highest of all, is relatively constant, and extends well beyond the previously defined 3′ ENOR boundary. Our previous experience using primer pairs against different positions of the same protein-coding genes had indicated that the expected differences in transcript copy number are generally less than 2-fold for the same transcript (unpublished data). Assuming such results can be applied here, the roughly 10-fold variation in CNS expression across ENOR28 challenges our original hypothesis of a single promoter driving expression across the entire region. Rather, it is possible that a number of separate transcripts are present, the longest of which spans primer pairs 1–11 and appears to be larger than 100 Kb. Interestingly, testis expression fell below detection threshold at both the 5′ and 3′ ends of ENOR28, suggesting the existence of a shorter testis-specific transcript.

Figure 4C shows that expression in the ENOR31 locus was relatively constant and extensive, with transcript copy numbers in CNS greater than 1,000 per 12.5 ng of total RNA not only within but also up- and downstream of the original ENOR boundaries. Approximately 10-fold expression spikes at primer pairs 15–16 and 7–8 suggested the possibility of up to three separate transcripts larger than 50 Kb. Testis expression gave a similar pattern but was much lower than CNS expression. Overall, then, assuming that a 10-fold variation in transcript levels between primer pairs is indicative of separate transcripts, both the ENOR28 and ENOR31 loci appear to produce not one but several macro ncRNAs (all of which are enriched in brain). However, it is worth noting that our data for *Air* (Figure 4A) (excluding regions with assembly gaps) also showed ~10-fold variation in transcript levels (e.g., primer pairs 9–10). Since *Air* is generally acknowledged to be a continuous transcript spanning ~108 Kb [5], it seems plausible that a 10-fold variation in transcript levels between primer pairs need not indicate multiple transcripts. If that is true, then the data for ENOR28 and ENOR31 would support the alternative conclusion that each region gives rise to a single macro ncRNA larger than 100 Kb.

### ENOR Transcripts Predominantly Localize to the Nucleus

**Figure 7:**
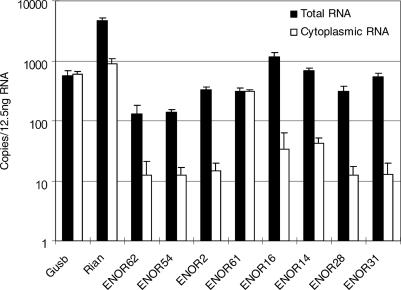
Localization of ENOR Transcripts qRT-PCR was carried out using total and cytoplasmic RNA from mouse whole brain and the corresponding primer pairs (Table S3). ENORs are listed in increasing order based on the estimated length of each region. Apart from the results shown, we also examined the localization of other mRNAs *(β-actin* and *GAPDH)* and additional regions of *Rian* and other ENORs, and these results were consistent with the rest (unpublished data).

Subcellular localization may provide clues to the function of ENOR transcripts. For instance, *Xist* exerts its chromosomal silencing effect within the nucleus [6]. We therefore examined the localization of the same eight ENORs (ENOR2, ENOR14, ENOR16, ENOR28, ENOR31, ENOR54, ENOR61, and ENOR62) we previously had characterized via northern blot by comparing brain expression levels from cytoplasmic and total RNA (the latter consists of both cytoplasmic and nuclear RNA). To validate our method, we initially tested β-glucuronidase *(Gusb)* mRNA, a housekeeping gene, and the *Rian* ncRNA (ENOR44), which preferentially localize to the cytoplasm and nucleus, respectively [28]. Figure 7 shows that, in keeping with our expectations, the copy number for *Gusb* mRNA was similar in cytoplasmic and total RNA (which suggests that there is a negligible nuclear RNA component) while *Rian* exists in cytoplasmic RNA at much lower levels than in total RNA (which suggests that the nuclear component predominates). Interestingly, when we examined the eight ENOR transcripts in an identical manner (Figure 7), seven of them (ENOR2, ENOR14, ENOR16, ENOR28, ENOR31, ENOR54, and ENOR62) showed much higher expression in total RNA, suggesting that they are localized in the nucleus. ENOR 61, on the other hand, appeared to be cytoplasmic.

## Discussion

The analysis of full-length enriched cDNA libraries has been of vital importance in improving our understanding of the mammalian transcriptome. In this regard, however, unspliced noncoding cDNAs are often viewed with skepticism because they can arise as truncation artifacts of cDNA library construction. Here, we have shown that such artifacts cluster within very long, functionally important ncRNAs such as *Air* and *Xist,* and, rather than summarily dismissing these cDNAs as worthless, we have employed a strategy that uses them to identify long ncRNAs genome-wide. The resulting list of 66 candidate ENORs—itself almost certainly an underestimate—potentially expands several-fold the number of known mouse ncRNAs larger than 10 Kb in size, which, to date, includes only a few examples such as *Xist, Air, Kcnq1ot1,* and *Ube3a-ats,* most of which were successfully detected with our methods. In the past, such macro ncRNAs have been discovered experimentally on an ad hoc basis, and it has not been possible to systematically identify large ncRNAs by bioinformatics means, since most existing tools are limited to the discovery of smaller ncRNAs with conserved primary sequences and/or secondary structures [44]. Our strategy offers a solution to this problem.

Expression studies produced a number of interesting observations. First, in silico analysis indicated that some ENORs cluster together within the genome and are coexpressed. For example, ENOR22 and ENOR23 are located within 2,300 Kb of each other on Chromosome 7 and are specifically expressed in CNS. One possible explanation for this coexpression is that these regions share a common chromatin domain. Second, we found that the majority of ENOR transcripts were predominantly nuclear, similar to functional ncRNAs such as *Xist* and *Tsix*. ncRNAs like these are increasingly being recognized as important in altering chromatin structure [45,46], and it is tempting to speculate that the ENOR transcripts might also function in this way. Third, qRT-PCR studies of the ENOR28 and ENOR31 loci (Figure 4) indicated that the actual transcribed regions are almost certainly underestimated based upon current ENOR boundaries. This is not surprising, since the boundaries were estimated using internally primed transcript coordinates, and reflects that our discovery pipeline was not designed to capture transcription start and end sites. Lastly, despite the possible existence of multiple macro ncRNAs in ENOR28 and ENOR31, expression correlation between the individual cDNAs was extremely high (average *R* = 0.96). This indicates that even if there are separate transcripts arising from each region they appear to be under the influence of similar regional promoters, enhancers, or chromatin domains. Fluorescence in situ hybridization studies might prove useful to visualize the ENOR transcripts and their surrounding chromatin structure (via the use of histone-specific antibodies), and may also directly demonstrate in which specific cell types and subcellular compartments ENORs are localized. For instance, knowing exactly which groups of neurons in the brain express ENOR28 and ENOR31 transcripts might provide indirect information as to their function. Understanding how the expression of these transcripts is regulated will also be important. For instance, fine-detailed mapping of transcript copy number by qRT-PCR using more primer pairs might better define the relevant transcriptional start sites and promoter regions.

Macro ncRNAs can function in a variety of ways, and some clues to the possible function of the ENORs can be gleaned from their association with antisense transcription, candidate imprinting domains, and miRNAs. Antisense transcripts exert regulatory effects in a number of ways, as mentioned earlier. Some of these effects (e.g., RNA interference and translation regulation) can be mediated by small miRNAs and siRNAs, and it is unclear if longer antisense transcripts—such as those identified in this study—are required to function in certain regulatory contexts. Of course, long antisense transcripts might be processed into smaller functional RNAs, although there has been no evidence that *Xist* or *Air,* for instance, work in this manner. Macro ncRNAs can also regulate genomic imprinting. *Ube3a-ats, Kcnq1ot1,* and *Air* have all been implicated in the imprinting control of their antisense transcripts. These three ncRNAs are themselves imprinted, a fact correctly predicted by the methods we used here. These same methods suggest that a further nine ENORs might represent potentially imprinted ncRNAs, which, if confirmed, would add substantially to the number of imprinted ncRNAs currently characterized. Finally, in silico analysis detected overlap between ENORs and more than 5% of known mouse miRNAs, suggesting that one of the possible functions of some of these regions may be to act as miRNA host genes. Given a recent report indicating that many mammalian miRNAs are still to be discovered [47], the possibility exists that more ENORs will be associated with novel miRNAs in the future.

Lacking any direct evidence of ENOR function, we also acknowledge the possibility that some of these regions do not play any functional role as RNAs. It has been shown, for instance, that expression of the yeast noncoding RNA *Srg1* is necessary for the repression of its downstream gene, *Ser3,* but this appears to be due to the act of *Srg1* transcription (causing *Ser3* promoter interference) rather than any direct action of the *Srg1* RNA itself [48]. Meanwhile, Wyers et al. found that intergenic transcripts in yeast are rapidly degraded by a specific nuclear quality control pathway and are therefore likely to be nonfunctional [49]. Another recent report in which megabase deletions of noncoding DNA were engineered and failed to produce any detectable phenotype in mice [50] suggests that large noncoding regions of the genome may not have function. It should be noted, however, that the regions targeted in this deletion study lacked evidence of transcription, in direct contrast to the regions we have characterized. A suggestion has also been made that many noncoding transcripts simply represent useless by-products of “leaky transcription” [51]. Based upon our expression studies of ENOR28 and ENOR31, transcripts from both these regions appear to be clearly expressed in brain (estimated at 1–8 copies/cell based upon our previous work [52], which is similar to *Air* [Figure 4] and to most mRNAs [53]), suggesting that in these cases, at least, transcripts are controlled. To demonstrate the importance (or otherwise) of the ENORs, it will ultimately be necessary to test their function directly. This, together with efforts to better understand the gene structure, expression, and regulation of individual transcripts within each region, is the challenge that lies ahead.

## Materials and Methods

### Identification of known mouse ncRNAs within the FANTOM3 cDNA collection.

Non-sno, non-micro reference mouse ncRNA sequences were downloaded from RNAdb, a database of mammalian ncRNAs (http://jsm-research.imb.uq.edu.au/rnadb) [24]. BLASTN was used to assess the similarity between the 102,801 FANTOM3 cDNAs and the reference ncRNAs using an initial *E*-value cutoff of 0.01, and any resulting hits with 98% or greater identity across 90% or more of the length of either a query cDNA or reference ncRNA sequence were considered significant matches. Repetitive sequences were identified in the FANTOM3 sequences using the union of RepeatMasker (http://www.repeatmasker.org) and runnseg predictions, and BLAST options –F “m” –U T were used to ignore repeats in the seeding but not the extension stage of the alignment.

### Genome-wide search for clusters of internally primed cDNAs.

We used the TU data prepared for the FANTOM3 project (ftp://fantom.gsc.riken.jp/RTPS/fantom3_mouse/primary_est_rtps/TU), which were generated by clustering the following mouse cDNA and EST sequences: (1) 56,006 mRNA sequences from GenBank (Release 139.0 and daily [2004–1–27]), (2) 102,597 RIKEN cDNAs from the FANTOM3 set, (3) 606,629 RIKEN 5′-end ESTs (5′-end set), (4) 907,007 RIKEN 3′-end ESTs (3′-end set), and (5) 1,569,444 GenBank EST sequences. Figure 2 summarizes the subsequent search pipeline, a full description of which was provided in the Results.

### Bioinformatic analysis of candidate clusters.

To judge whether ENOR sequences were spliced or unspliced, we searched for all TUs that overlapped with the chromosomal boundaries of each ENOR and were on the same strand. We included any spliced TUs whose intronic area overlapped with a region. We then counted ESTs associated with the TUs and classified the regions as follows: spliced, if spliced ESTs were more than 10% of total ESTs; otherwise unspliced. We used the threshold of 10% since a certain number of ESTs can be expected to be inappropriately mapped onto the genome and may therefore appear as falsely spliced ESTs. To find transcripts on the sense or antisense strand, we searched for TUs that overlapped with the regions on the same or opposite strand based on genomic coordinates. We searched for the gene name associated with these TUs, as defined by the RTPS pipeline used for FANTOM2 and FANTOM3 [54], and selected appropriate names manually. For the spliced ENORs, we selected the gene name of major transcripts on the same strand. For the unspliced ENORs, we used only informative gene names because uninformative names such as the RIKEN clone IDs were associated with unspliced cDNAs that covered only short regions. We also searched for gene names on the MGI database (http://www.informatics.jax.org) and used official gene symbols if available.

To examine ENOR conservation, we used blastz axtNet alignments from UCSC (ftp://hgdownload.cse.ucsc.edu/goldenPath/mm5/vsHg17) to identify blocks in the mouse genome that successfully align with the human genome. We classified individual ENORs as conserved if the total length of alignable blocks was greater than 50% of the ENOR length. To determine the overall conservation levels of ENOR sequences, the mouse genome was divided into 50-nucleotide windows, and the number of identically matching nucleotides in each window in the human genome was counted for both the ENORs and the genome as a whole.

Information on candidate imprinted cDNAs was provided by Nikaido et al. ([38]; http://fantom2.gsc.riken.go.jp/imprinting), and lists of miRNA and snoRNAs were downloaded from the miRBase Registry (http://www.sanger.ac.uk/Software/Rfam/mirna) and RNAdb, respectively, and mapped to the mouse genome (mm5) using MEGABLAST with options –F F –D 1 –J F. We then searched these imprinted cDNAs, miRNAs, and snoRNAs for overlap with the ENOR loci based on genomic coordinates. To determine whether the association between candidate imprinted cDNAs and ENOR loci was likely to have occurred by chance, we randomly sampled 66 regions with an average cDNA density equal to that of the ENORs (five RIKEN cDNAs per region) and determined the number of regions that contained at least one candidate imprinted cDNA; this procedure was repeated 100 times, and the significance was determined using a Chi-square test. To determine the significance of the association between miRNAs and ENOR loci, we performed the following calculation: given that ENORs cover 0.23% of the genome, the probability that a miRNA lies in an ENOR on the same strand is 0.0023 × 0.5. Using the binomial distribution, the probability that 14 or more out of 224 miRNAs lie in ENORs is about 3 × 10^−20^ (i.e., *p* < 0.0001).

To examine ENOR expression, we identified GNF Gene Expression Atlas (http://expression.gnf.org/cgi-bin/index.cgi) probes that overlapped with the genomic loci of the ENORs via the UCSC Genome Browser (http://genome.ucsc.edu), then downloaded the relevant expression data (http://symatlas.gnf.org). Affymetrix MAS5 software absent/present calls were used to identify probes with detectable expression in at least one of the 61 tissues tested. Log 2 ratio expression data for these probes were then hierarchically clustered via average linkage clustering using Cluster software [55]. Additionally, we downloaded the list of RIKEN libraries and their corresponding Edinburgh Mouse Atlas Project (http://genex.hgu.mrc.ac.uk) tissue descriptions, then searched for RIKEN ESTs that mapped within an ENOR region on the same strand, and tallied the number of ESTs that were derived from each tissue library. We counted 5′ EST and 3′ EST sequences derived from a same clone only once. Library information for some ESTs could not be used because of uninformative tissue descriptions.

### Primers.

Primer pairs were designed using Primer3 software [56], with an optimal primer size of 20 bases and annealing temperature of 60 °C (see Table S3). The uniqueness of the designed primer pairs was checked by a BLAST search (http://www.ncbi.nlm.nih.gov/BLAST) so that homologous regions were not cross-amplified by the same primer pair.

### Preparation of RNA samples.

Adult male C57BL/6J mice were killed according to the RIKEN Institute's guidelines, and the tissues were removed. Total RNA was extracted by the acid phenol-guanidinium thiocyanate-chloroform method [57]. Cytoplasmic RNA was prepared as described elsewhere [58]. RNA was checked by agarose gel electrophoresis and was treated with DNaseI before RT-PCR as described elsewhere [52].

### RT-PCR analysis of candidate clusters.

First-strand cDNA synthesis (5 μg of total RNA per 20-μl reaction) was carried out using a random primer and the ThermoScript RT-PCR System (Invitrogen; http://www.invitrogen.com) in accordance with the manufacturer's protocol. qRT-PCR was carried out with first-strand cDNA corresponding to 12.5 ng of total RNA per test well using the tailor-made reaction [52]. The PCR reactions were performed with an ABI Prism machine (Applied Biosystems; http://www.appliedbiosystems.com) using the following cycling protocols: 15-min hot start at 94 °C, followed by 40 cycles of 15 s at 94 °C, 30 s at 60 °C, and 30 s at 72 °C. The threshold cycle (Ct) value was calculated from amplification plots, in which the fluorescence signal detected was plotted against the PCR cycle. The number of transcripts was calculated from the slope of the standard curve using genomic DNA.

### Long PCR.

Long PCR was carried out with first-strand cDNA corresponding to 500 ng of total RNA and KOD DNA polymerase (Toyobo; http://www.toyobo.co.jp/e) per 50-μl reaction according to the manufacturer's protocol. We also used 200 ng of mouse genomic DNA, instead of first-strand cDNA, to amplify the fragments from the genome. The PCR reactions were performed with an ABI9700 (Applied Biosystems) using the following cycling protocols: 2-min hot start at 94 °C, followed by 35 cycles of 15 s at 94 °C, 30 s at 60 °C, and 5 min at 68 °C. One to two microliters of sample was subjected to 1% agarose gel electrophoresis.

### Northern blot.

Total RNA was denatured by formaldehyde/formamide and electrophoresed in a 1% agarose gel. RNA was transferred onto Hybond-N+ nylon membrane (GE Healthcare Life Sciences; http://www4.amershambiosciences.com). Hybridization was carried out using ^32^P-labeled DNA probe and ExpressHyb hybridization solution (BD Biosciences; http://www.bdbiosciences.com) according to the manufacturer's protocol. The hybridization signal was detected using a BAS2500 image analyzer (Fujifilm; http://www.fujifilm.com).

## Supporting Information

Figure S1 Genomic Distribution of 66 ENORs (68 KB PPT)

Figure S2 ENORs Are More Conserved than the Genome Average (68 KB PPT)

Table S1 ENORs on Each Chromosome (17 KB XLS)

Table S2 EST Tissue Data for 66 ENORs (33 KB XLS)

Table S3 Primer Pairs (39 KB XLS)

Table S4 Expression Correlation between cDNAs within ENOR28 and ENOR31 Data for (A) ENOR28 and (B) ENOR31.
(19 KB XLS)

### Accession Numbers

The MGI (http://www.informatics.jax.org) accession numbers for the sequences described in this paper are *3222402P14Rik* (2442104), *4933421G18Rik* (1913976), *Air* (1353471), *Cyp2d22* (1929474), *Dleu2* (1934030), *Dmd* (94909), *Emx2os* (3052329), *Gusb* (95872), *Igf2r* (96435), *Kcnq1ot1* (1926855), *Kcnrg* (2685591), *Mcp* (1203290), *Ndufs1* (2443241), *Nespas* (1861674), *Nr6a1* (1352459), *Ppp2r5a* (1929474), *Rian* (19222995), *Satb1* (105084), *Slc22a2* (18339), *Slc22a3* (1333817), *Tgif* (1194497), *Traf3ip2* (2143599), *Trim13* (1913847), *Tsix* (1336196), *Ube3a* (105098), and *Xist* (98974). The SGD (http://www.yeastgenome.org) accession number for yeast *Srg1* is S000029010. The NCBI EntrezGene (http://www.ncbi.nlm.nih.gov/entrez/query.fcgi?db=gene) accession number for yeast *Ser3* is 856814.

## Abbreviations

CNS: central nervous system
ENOR: expressed noncoding region
EST: expressed sequence tag
GEV: FANTOM3 Genomic Element Viewer
GNF: Genomics Institute of the Novartis Research Foundation
Kb: kilobase(s)
miRNA: microRNA
ncRNA: non-protein-coding RNA
qRT-PCR: quantitative real-time RT-PCR
siRNA: short interfering RNA
snoRNA: small nucleolar RNA
TU: transcriptional unit
UCSC: University of California Santa Cruz
UNA: unspliced, noncoding, and containing adjunct adenine-rich regions
