# Supplementary material for: Clusters of Internally Primed Transcripts Reveal Novel Long Noncoding RNAs
Source: PLoS Genet. 2006 Apr 28;2(4):e37. doi: 10.1371/journal.pgen.0020037 (PMC1449886; doi:10.1371/journal.pgen.0020037)
Supplement: Figure S1 — (68 KB PPT) [file pgen.0020037.sg001.ppt]

## Slide 1
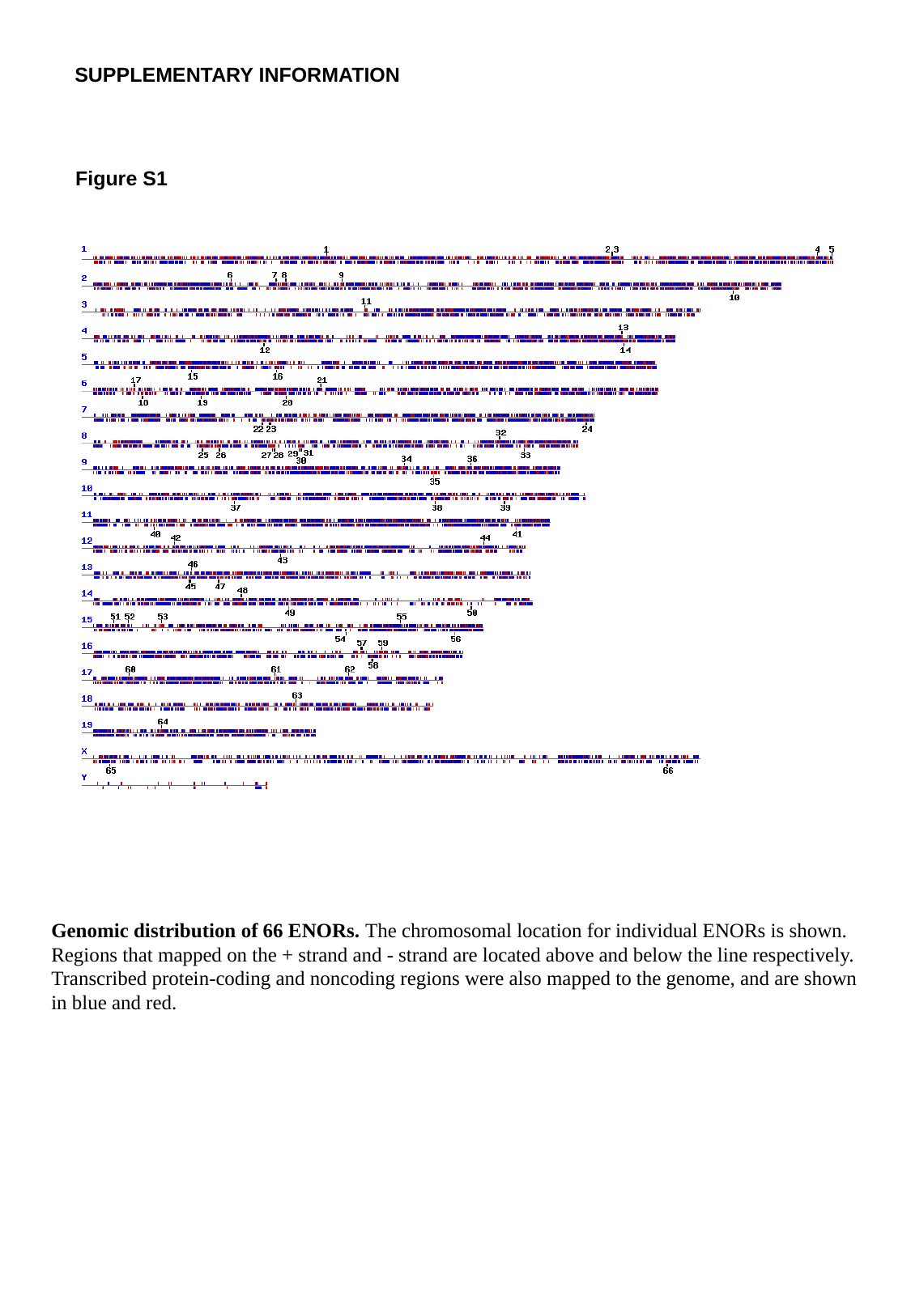

SUPPLEMENTARY INFORMATION
Figure S1
Genomic distribution of 66 ENORs. The chromosomal location for individual ENORs is shown. Regions that mapped on the + strand and - strand are located above and below the line respectively. Transcribed protein-coding and noncoding regions were also mapped to the genome, and are shown in blue and red.
