# Supplementary material for: Clusters of Internally Primed Transcripts Reveal Novel Long Noncoding RNAs
Source: PLoS Genet. 2006 Apr 28;2(4):e37. doi: 10.1371/journal.pgen.0020037 (PMC1449886; doi:10.1371/journal.pgen.0020037)
Supplement: Figure S2 — (68 KB PPT) [file pgen.0020037.sg002.ppt]

## Slide 1
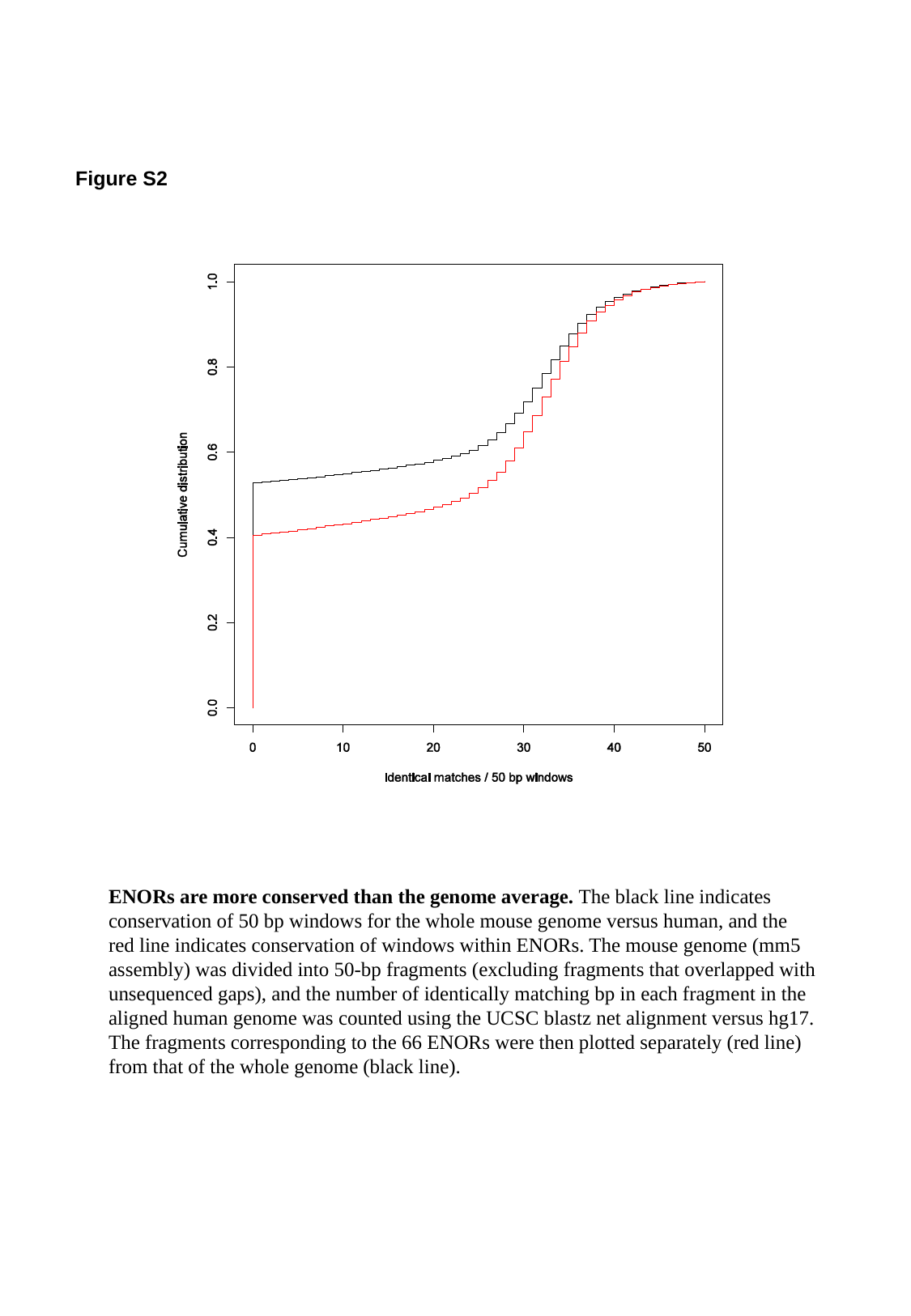

Figure S2
ENORs are more conserved than the genome average. The black line indicates conservation of 50 bp windows for the whole mouse genome versus human, and the red line indicates conservation of windows within ENORs. The mouse genome (mm5 assembly) was divided into 50-bp fragments (excluding fragments that overlapped with unsequenced gaps), and the number of identically matching bp in each fragment in the aligned human genome was counted using the UCSC blastz net alignment versus hg17. The fragments corresponding to the 66 ENORs were then plotted separately (red line) from that of the whole genome (black line).
